# A new *Liopropoma* sea bass (Serranidae, Epinephelinae, Liopropomini) from deep reefs off Curaçao, southern Caribbean, with comments on depth distributions of western Atlantic liopropomins

**DOI:** 10.3897/zookeys.409.7249

**Published:** 2014-05-15

**Authors:** Carole C. Baldwin, D. Ross Robertson

**Affiliations:** 1 Department of Vertebrate Zoology, National Museum of Natural History, Smithsonian Institution, Washington, DC 20560; 2 Smithsonian Tropical Research Institute, Balboa, Republic of Panamá

**Keywords:** *Liopropoma aberrans*, *Liopropoma olneyi*, submersible, Substation Curaçao, Deep Reef Observation Project (DROP), DNA barcoding, phylogeny, modes of speciation

## Abstract

Collecting reef-fish specimens using a manned submersible diving to 300 m off Curaçao, southern Caribbean, is resulting in the discovery of numerous new fish species. The new *Liopropoma* sea bass described here differs from other western Atlantic members of the genus in having VIII, 13 dorsal-fin rays; a moderately indented dorsal-fin margin; a yellow-orange stripe along the entire upper lip; a series of approximately 13 white, chevron-shaped markings on the ventral portion of the trunk; and a reddish-black blotch on the tip of the lower caudal-fin lobe. The new species, with predominantly yellow body and fins, closely resembles the other two “golden basses” found together with it at Curaçao: *L. aberrans* and *L. olneyi*. It also shares morphological features with the other western Atlantic liopropomin genus, *Bathyanthias*. Preliminary phylogenetic data suggest that western Atlantic liopropomins, including *Bathyanthias*, are monophyletic with respect to Indo-Pacific *Liopropoma*, and that *Bathyanthias* is nested within *Liopropoma*, indicating a need for further study of the generic limits of *Liopropoma*. The phylogenetic data also suggest that western Atlantic liopropomins comprise three monophyletic clades that have overlapping depth distributions but different depth maxima (3–135 m, 30–150 m, 133–411 m). The new species has the deepest depth range (182–241 m) of any known western Atlantic *Liopropoma* species. Both allopatric and depth-mediated ecological speciation may have contributed to the evolution of western Atlantic Liopropomini.

## Introduction

Submersible diving to 300 m off Curaçao in the southern Caribbean as part of the Smithsonian Institution’s Deep Reef Observation Project (DROP) is expanding our knowledge of the deep-reef Caribbean fish fauna (Baldwin and Robertson 2013, Baldwin and Johnson 2014). Recent collections of fishes included multiple individuals of what we initially identified as *Liopropoma aberrans* (Poey 1860) based on their predominantly golden color pattern. Subsequent analysis of mitochondrial DNA sequences (COI) from those specimens, detailed morphological examination of the preserved voucher specimens, and the discovery of consistent patterns of variation in coloration in photographs of vouchers taken prior to preservation led to the description of some of those individuals as a new species, *Liopropoma olneyi* Baldwin & Johnson, 2014. Additional genetic and morphological data indicate that the “golden basses” off Curaçao, in fact, comprise three species, *Liopropoma aberrans*, *Liopropoma olneyi*, plus one undescribed species. Herein we describe this third species, *Liopropoma santi* sp. n.

*Liopropoma* (Atlantic and Pacific), *Bathyanthias* (western Atlantic), and the monotypic *Rainfordia* (Indo-Pacific) form the monophyletic epinepheline serranid tribe Liopropomini (Baldwin and Johnson 1993). Twelve species of liopropomins currently are known from the western Atlantic, including the new species described herein: seven species of *Liopropoma*, four species of *Bathyanthias*, and a putative new species of the latter genus that we refer to here. These western Atlantic liopropomin species inhabit both shallow (< 50 m) and deep (to 411 m) reefs in Caribbean and adjacent waters. To compare species depth preferences, we use the known depth maximum and minimum for each species. To investigate how deep and shallow species are interrelated, we use the COI data to hypothesize the phylogeny of the group and then analyze the results in the context of the known depth distributions of the various species. Based on these results, we comment on possible modes of speciation in western Atlantic liopropomins.

## Materials and methods

The manned submersible *Curasub* (http://www.substation-Curacao.com) was employed to collect fishes and invertebrates during various field periods between 2011 and 2013. Fish specimens were collected using the fish anesthetic quinaldine pumped from a reservoir through a tube attached to one hydraulic arm of the sub and a suction hose (that uses the same pump as the anesthetic-delivery apparatus) attached to the other arm. The latter empties into a vented plexiglass cylinder attached to the outside of the sub. At the surface, the specimens were measured, photographed, tissue sampled (muscle biopsy from right side) and preserved. They were later x-rayed with a digital radiography system. Counts and measurements included in the description follow Hubbs and Lagler (1958) and Randall and Taylor (1988). Measurements were made to the nearest 0.1 mm with an ocular micrometer fitted into a Wild stereomicroscope (smallest specimen) or with needle-point dial calipers. Institutional abbreviations follow Sabaj Pérez (2012).

Tissue samples for DNA Barcoding were stored in saturated salt-DMSO (dimethyl sulfoxide) buffer (Seutin et al. 1991). DNA extraction, PCR, sequencing cytochrome c oxidase subunit I (COI), and editing COI sequences were performed as outlined by Weigt et al. (2012). A neighbor-joining tree (Saitou and Nei 1987) was generated using PAUP*4.1 (Swofford 2002) on an analysis of Kimura two-parameter distances (Kimura 1980). The neighbor-joining tree shows genetic distances in COI among individuals and how they cluster into genetically distinct lineages, which, in teleost fishes, correspond well with species (e.g. Baldwin and Weigt 2012, Weigt et al. 2012). Interspecific phylogenetic relationships were hypothesized for western Atlantic liopropomins and three Indo-Pacific species of *Liopropoma* based on maximum parsimony analysis of the COI sequences using heuristic searches in PAUP*4.1. Characters were equally weighted and left unordered. The resulting equally parsimonious trees were summarized using the strict consensus method. Outgroups for both analyses were two members of the sister group of the Liopropomini—*Grammistes sexlineatus* (Thunberg, 1782) and *Rypticus carpenteri* Baldwin & Weigt, 2012, of the tribe Grammistini (Baldwin and Johnson 1993), and the trees were rooted on a more distant outgroup, *Scorpaena plumieri* of the family Scorpaenidae. We follow Johnson (1983) and Baldwin and Johnson (1993) in recognizing a monophyletic family Serranidae and subfamily Epinephelinae pending resolution of serranid relationships in light of conflicting hypotheses based on molecular data (e.g., Smith and Craig 2007, Betancur et al. 2013, Near et al. 2013).

The label for each entry on the neighbor-joining tree is an assigned DNA number, and we include that number in the designation of type specimens and in some figure captions. Abbreviations used in DNA numbers are as follows: BAH–Bahamas, BLZ–Belize, CUR–Curacao, FLST–Florida Straits, FWRI–Florida Wildlife Research Institute, MBIO–Moorea Biocode Project, MCgroup–Matthew Craig, MOC–*Miguel Oliver* Caribbean Cruise, MOOP–Moorea Deep Reef, TOB–Tobago. GenSeq nomenclature for DNA sequences (Chakrabarty et al. 2013) and GenBank information are presented along with museum catalog numbers for voucher specimens in the Appendix.

## Results

**Figure 1.**
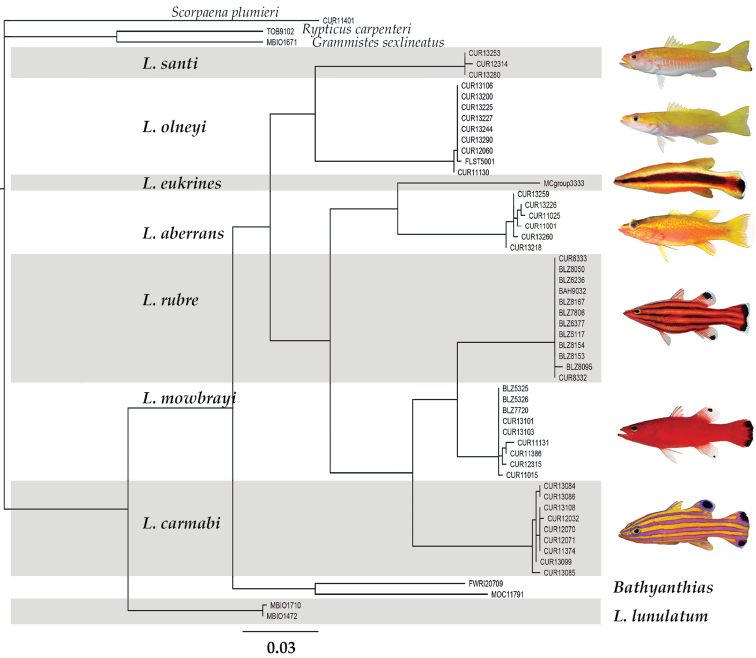
Neighbor-joining tree derived from COI sequences for western Atlantic *Liopropoma*, the Indo-Pacific *Liopropoma lunulatum*, and related taxa. The tree was rooted on *Scorpaena plumieri*. Divergence represented by scale bar = 3%. Photographs of *Liopropoma rubre* and *Liopropoma mowbrayi* by James Van Tassell and Ross Robertson.

The neighbor-joining tree (Fig. 1) shows how individual specimens of western Atlantic *Liopropoma* sort into genetic lineages based on similarity in COI sequences. Lineages correlate well with currently recognized species. Genetic distance in COI between pairs of species of western Atlantic *Liopropoma* ranges from 5–18%, and distance between *Liopropoma santi* sp. n., and other western Atlantic *Liopropoma* species is 13–18% (Table 1). Average intraspecific variation for western Atlantic *Liopropoma* is 0–0.3%, 0.2% for *Liopropoma santi*.

**Table 1.** Average (and range) Kimura two–parameter distance summary for species of western Atlantic *Liopropoma* (7), Indo–Pacific *Liopropoma* (1), western Atlantic *Bathyanthias* (2), and outgroups *Grammistes*, *Rypticus*, and *Scorpaena* based on cytochrome c oxidase I (COI) sequences of individuals represented in the neighbor–joining tree in Figure 1. Intraspecific averages are shown in bold. “na” = not applicable (n=1).

	*Liopropoma aberrans*	*Liopropoma carmabi*	*Liopropoma eukrines*	*Liopropoma lunulatum*	*Liopropoma mowbrayi*	*Liopropoma olneyi*	*Liopropoma rubre*	*Liopropoma santi* sp. n.	*Bathyanthias mexicanus*	*Bathyanthias* sp.	*Grammistes sexlineatus*	*Rypticus carpenteri*	*Scorpaena plumieri*
	W. Atl.	W. Atl.	W. Atl.	Indo-Pacific	W. Atl.	W. Atl.	W. Atl.	W. Atl.	W. Atl.	W. Atl.	Indo-Pacific	W. Atl.	W. Atl.
	(n=6)	(n=9)	(n=1)	(n=2)	(n=9)	(n=9)	(n=12)	(n=3)	(n=1)	(n=1)	(n=1)	(n=1)	(n=1)
*Liopropoma aberrans*	**0.3 (0–0.6)**												
*Liopropoma carmabi*	14.6 (14.2–15.2)	**0.2 (0–0.6)**											
*Liopropoma eukrines*	10.5 (10.2–10.8)	15.1 (14.8–15.6)	**na**										
*Liopropoma lunulatum*	14.8 (14.6–15.1)	16.4 (16.1–16.9)	14 (14.0–14.1)	**0.1 (0–0.2)**									
*Liopropoma mowbrayi*	12.2 (11.9–12.5)	8.6 (8.2–9.1)	13.5 (13.4–13.9)	15.5 (15.3–15.8)	**0.2 (0–0.6)**								
*Liopropoma olneyi*	11.8 (11.5–12.1)	13.6 (13.4–14.2)	13 (12.8–13.1)	14.7 (14.4–14.9)	13.3 (13.0–13.7)	**0 (0–0.3)**							
*Liopropoma rubre*	11.9 (11.5–12.4)	10.5 (10.1–10.9)	12.9 (12.8–13.3)	15.8 (15.3–16)	5.7 (5.3–6.0)	12.5 (12.2–13.2)	**0 (0–0.3)**						
*Liopropoma santi* sp. n.	16.2 (16.0–16.7)	17.6 (17.1–18.4)	15.2 (15.0–15.6)	16.4 (16.0–16.9)	15.5 (14.8–16.5)	13.3 (13.0–13.5)	16.4 (15.9–17.6)	**0.2 (0.0–0.3)**					
*Bathyanthias mexicanus*	16.1 (15.8–16.4)	17.6 (17.1–18.4)	15.2 (15.0–15.6)	16.4 (16.0–16.9)	13.9 (13.8–14.1)	13.4 (13.4–13.5)	13.8 (13.7–14.3)	16.2 (15.9–16.9)	**na**				
*Bathyanthias* sp.	16.8 (16.5–17)	15.4 (15.2–15.7)	15.4 (-)	16 (15.9–16.1)	14.8 (14.6–14.9)	15.2 (15.1–15.4)	14.6 (14.5–14.8)	16.4 (16.2–16.7)	13.7 (-)	**na**			
*Grammistes sexlineatus*	18.6 (18.3–18.8)	17.9 (17.7–18.4)	18 (-)	17 (16.9–17.1)	18.1 (18.0–18.5)	18.9 (18.7–18.9)	18 (17.8–18.3)	20.8 (20.5–21.5)	19.8 (-)	15.9 (-)	**na**		
*Rypticus carpenteri*	17.3 (17.1–17.5)	17.9 (17.9–18.1)	14.8 (-)	15.4 (15.4–15.5)	16.8 (16.6–17)	18.6 (18.4–18.6)	17.4 (17.2–17.5)	17.5 (17.5–17.6)	18.9 (-)	16.8 (-)	13.2 (-)	**na**	
*Scorpaena plumieri*	21.4 (21.2–21.5)	21.6 (21.4–22)	20.9 (-)	19.8 (19.7–19.8)	19.7 (19.5–20.4)	20.8 (20.8)	20.3 (20.2–20.6)	24.5 (24.4–24.8)	19.2 (-)	20.7 (-)	19.5 (-)	19.6 (-)	**na**

### Liopropoma santi sp. n.

http://zoobank.org/83D20375-39CA-457D-8D54-127ACC3ED0B7

http://species-id.net/wiki/Liopropoma_santi

**Figure 2.**
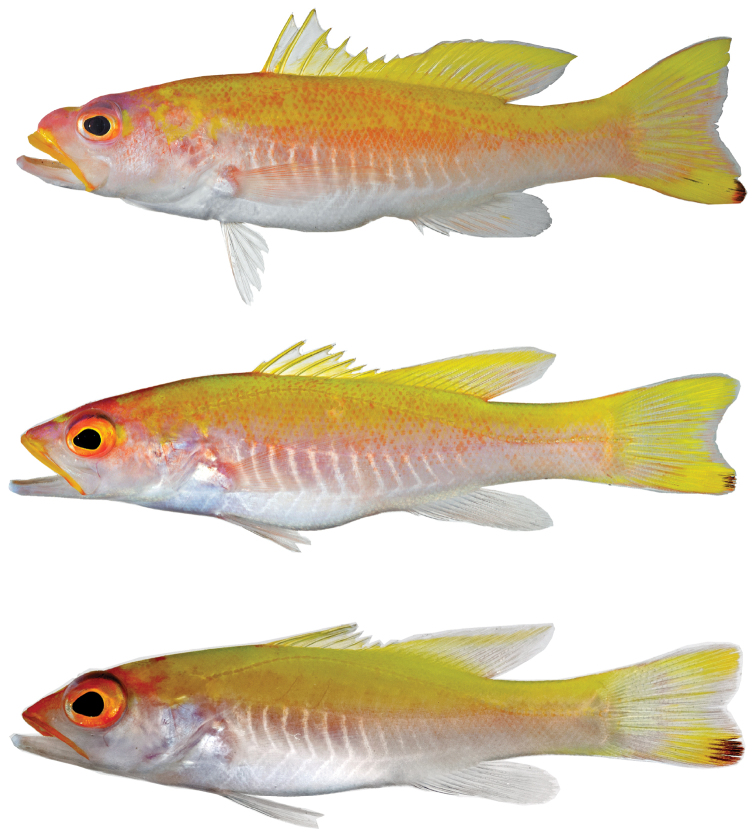
*Liopropoma santi* sp. n., type series: **A** USNM 426811, holotype, 116 mm SL, DNA #CUR 13253 **B** USNM 426813, paratype, 76.2 mm SL, DNA #CUR 13280 **C** USNM 414824, paratype, 42.0 mm SL, DNA #CUR 12314.

**Figure 3.**
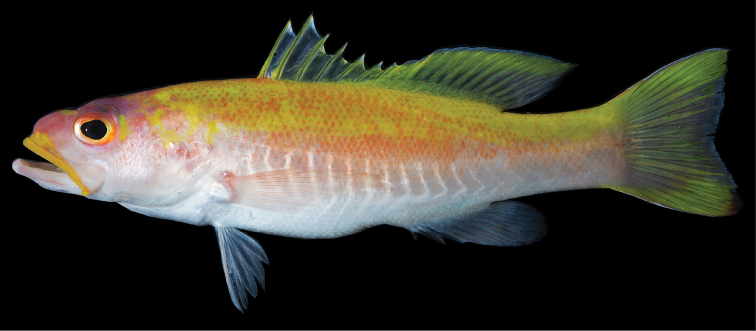
*Liopropoma santi* sp. n., USNM 426811, holotype, 116 mm SL (photographed against a black background).

**Figure 4.**
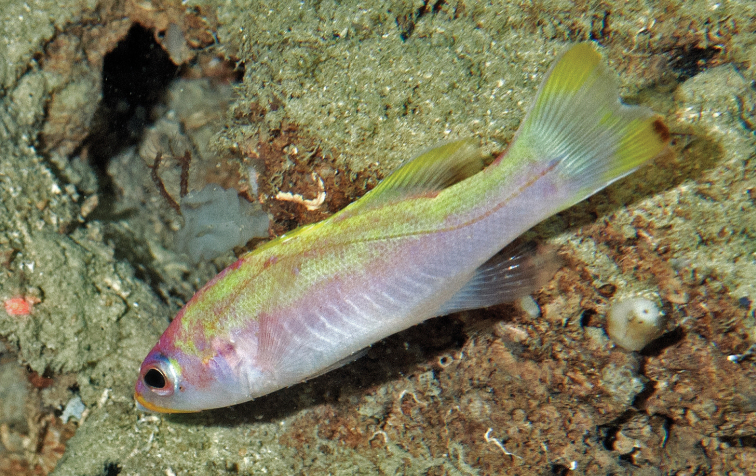
In-situ photograph of *Liopropoma santi* sp. n., taken from the *Curasub* submersible at 204 m on a reef slope off Jan Theil Bay, Curaçao, 5 Nov 2013. Photo courtesy of Substation Curaçao.

Figs 2 – 4 , Spot-tail Golden Bass

#### Type locality.

Curaçao, southern Caribbean

#### Holotype.

USNM 426811, 116 mm SL, DNA #CUR 13253, *Curasub* submersible, sta. 13-14, southern Caribbean, Curaçao, off Substation Curaçao downline, near 12°05.069'N, 68°53.886'W, 241 m, quinaldine, 9 Aug 2013, C. C. Baldwin, D. R. Robertson, A. Driskell, B. van Bebber.

**Paratypes.** USNM 426813, 76.2 mm SL, DNA #CUR 13280, *Curasub* submersible, sta. 13–19, southern Caribbean, Curaçao, Playa Forti, Westpoint, 12°22.001'N, 69°9.005 W, 182 m, quinaldine, 15 Aug 2013, A. Schrier, N. Knowlton, R. Sant, B. van Bebber. USNM 414824, 42.0 mm SL, DNA #CUR 12314, *Curasub* submersible, sta. 12–19, southern Caribbean, Curaçao, east of Substation Curaçao downline, near 12°05.069'N, 68°53.886'W, 209 m, 15 Aug 2012, C. C. Baldwin, B. Brandt, B. van Bebber.

#### Diagnosis.

A liopropomin serranid with the following combination of characters: dorsal fin VIII,13; anal fin III, 8; pectoral fin 15; total gill rakers on first arch (including rudiments) 20–21; lateral-line scales 47–48; length of first dorsal spine 2.9–4.2% SL; margin of spinous dorsal fin moderately indented posteriorly in adults (fourth spine 11–12% SL, fifth and sixth spines only slightly shorter than fourth—6.9–10% SL); depth at origin of dorsal fin 23–26% SL; least depth of caudal peduncle 11–13% SL; orbit diameter 9.4–12% SL; yellow-orange stripe externally on upper lip; series of approximately 13 white, chevron-shaped markings on ventral portion of trunk; reddish-black blotch on distal portion of lower caudal-fin lobe; inhabiting depths of 182–241 m.

#### Description.

Counts and measurements of holotype, if different from those of paratypes, are given in parentheses. Dorsal-fin rays VIII, 13; anal-fin rays III, 8; pectoral-fin rays (both sides) 15; pelvic-fin rays I, 5; principal caudal-fin rays 9+8=17; procurrent caudal-fin rays 9+9=18; pored lateral-line scales 48 (47), two additional pored scales present on base of caudal fin not included in total count; scales from lateral line to dorsal-fin origin 3 or 4 (3); gillrakers on first arch, including rudiments, 6+14-15 (6+14); upper limb with 3 rudiments + 3 rakers, lower limb with 11-13 rakers + 2-3 rudiments, total 20–21 (20); vertebrae 10 + 14.

Body proportions expressed as percentage of SL. Body depth at origin of dorsal fin 23–26 (26); body width just behind gill opening 11–14 (14); head length 37–39 (37); snout length 7.4–9.1 (9.1), relative length increasing with increasing SL; orbit diameter 9.4–12 (9.4) relative diameter decreasing with increasing SL; bony interorbital width 4.5–5.5 (5.5); upper-jaw length 16–18 (18); greatest depth of maxilla 5.0–6.1 (6.1); least caudal-peduncle depth 11–13 (13); caudal-peduncle length 22–24 (23); lengths of dorsal-fin spines: (I) 2.9–4.2 (4.2); (II) 11–12 (12); (III) 13–15 (14); (IV) 11–12 (11); (V) 6.9–10 (10); (VI) 6.9–8.2 (8.2); (VII) 5.0–7.5 (7.5); (VIII) 4.8–6.9 (6.9); longest dorsal soft ray the 11^th^, length 15–20 (20); length of 3^rd^ anal-fin spine 6.9–9.3 (9.3); longest anal soft ray the 5^th^, length 15–17 (16); caudal-fin length 23–28 (23), relative length decreasing with increasing SL; pectoral-fin length 27–30 (27), fin reaching vertical between anus and origin of anal fin, falling short of anal fin in all specimens; pelvic-fin length 18–20 (19), fin reaching vertical through base of 6^th^ dorsal-fin spine, well short of anus.

Interorbital region flat to slightly convex; mouth oblique, maxilla reaching vertical beyond posterior border of pupil; prominent bony projection on posteroventral corner of maxilla; lower jaw slightly projecting. Anterior nostril in thin, membranous tube, nostril situated just posterior to groove between tip of snout and premaxilla; posterior nostril a simple opening, nostril situated close to orbit (the distance approximately 1.5 nostril diameters). Lateral line strongly arched above pectoral fin, highest point below fourth and fifth dorsal-fin spines.

Trunk covered with ctenoid scales, scales becoming weakly ctenoid anteriorly and cycloid on head. Head fully scaled except over branchiostegal area. Holotype with short column of scales on dorsal-fin spines III and IV, scales on basal portion of membranes between spines VI and VIII, three rows of scales covering basal portion of soft dorsal fin, and some scales extending distally onto soft dorsal-fin membranes; paratypes with same squamation except no scales present on spinous dorsal fin, and 42.0-mm SL paratype having only basal scale rows on soft dorsal fin. In holotype and larger paratype, anal fin with two or three rows of scales basally and additional scales that extend distally onto fin membranes and cover most of fin. In smaller paratype, scales confined to basal portion of fin. Caudal fin completely scaled in holotype except for distal tips of rays; larger paratype with scales covering only proximal half of fin; smaller paratype with scales confined to basal portion of fin. Scales present on pectoral-fin base, and elongate scales present on proximal portion of fin. Scales present on pelvic-fin base and on proximal portion of fin; pelvic axillary scales present.

Jaw teeth small and depressible; upper and lower jaws with bands of villiform teeth, bands widest anteriorly, largest teeth in innermost row. Vomer with a chevron-shaped patch of small teeth. Palatines with several rows of small teeth in a long, narrow band. Opercle with three flattened spines, only the middle one conspicuous. Margin of upper limb of preopercle and angle with small serrations, lower limb smooth.

Prior to preservation (Figs 2, 3), background color of upper portions of trunk and caudal peduncle yellow, grading to pale pink around midbody, then to white ventrally; no abrupt transitions between those colors; many individual scales on upper half of body marked with orange spots in adults, densely so in holotype; a series of about 13 narrow, bright-white, chevron-shaped bars that point posteriorly present on lower half of trunk, series extending from just behind pectoral-fin base to vertical through center or posterior portion of anal fin; upper arms of white bars more strongly defined; nape yellow from dorsal midline ventrally to about mid-eye level (with some orange spots on scales in adults), grading anteriorly into an irregularly shaped area of purplish-pink over and behind eye, on upper portion of iris, and on snout; a yellow blotch present behind center of eye (in adults) and a smaller one present on dorsal midline of snout just anterior to orbit; iris mostly orange-yellow, grading to fine inner yellow ring; prominent, mostly deep-yellow (adults) or mostly orange (juvenile) stripe along outside of entire upper lip, this pigment spreading slightly above lip along anterior half of jaw in adults and merging with the pink/orange pigment on snout of juvenile; inside of lower lip with small blotch of yellow pigment in adults, inside of upper lip with stripe of yellow (adults) or orange (juvenile); photographic angle did not permit characterization of pigment on inside of lower lip of juvenile; lower jaw and lower two thirds of head white, with pinkish cast in holotype; in adults, dorsal fin with yellow spines and mostly white inter-spinous membranes; soft dorsal-fin rays yellow, membrane between anterior rays yellow, and membrane between rays of remainder of fin with small to large pale area centrally, size of pale area increasing posteriorly such that membrane between posteriormost rays completely pale; some rays and membranes in posterior portion of soft dorsal fin with pale rose pigment in smaller adult; a thin white margin extending along outer edge of entire dorsal fin, this margin appearing blue-white when fish photographed against black background (Fig. 3); in juvenile, inter-spinous membranes of dorsal fin mostly pale and soft dorsal mostly pale except for yellow stripe at the base and yellow stripe near outer margin of fin; caudal fin mostly yellow in holotype, central portion of fin with pale outer margin and with pale to pinkish-orange membranes between rays; thin pinkish-orange stripe present along dorsal and ventral margins of fin; distal tip of lower lobe with reddish-black blotch, a few thin streaks of black extending proximally from this blotch; pigment on caudal fin of smaller adult similar but with less pinkish-orange pigment, and caudal fin of juvenile mostly clear with a large, oval-shaped, oblique yellow blotch on outer half of both upper and lower lobes; dark spot on distal portion of ventral caudal lobe relatively larger in juvenile; anal fin white, with faint pinkish-yellow streak on first through fifth rays in holotype, little or no color in smaller adult and juvenile; pelvic fin white; pectoral fin translucent with pale pinkish-orange cast; general coloration most intense in the holotype and least intense in the juvenile.

In alcohol (see Fig. 6A), body pale, the only pigment a dark blotch on distal tip of ventral caudal-fin lobe.

#### Distribution.

Known only from Curaçao, southern Caribbean.

#### Habitat.

Off Curaçao, *Liopropoma santi* is found from 182–241 m inhabiting rocky slopes and ledges. It retreats into small caves and crevices when approached and illuminated by the submersible. Figure 4 shows an in-situ photograph taken from the *Curasub* submersible at 204 m on a reef slope off Jan Theil Bay, Curaçao.

#### Etymology.

The specific name honors Roger Sant, who participated in the *Curasub* submersible dive at Playa Forti during which the USNM 426813 paratype was collected. Roger and Victoria Sant have provided generous funding to the Smithsonian Institution’s National Museum of Natural History for ocean-related activities.

#### Common name.

“Spot-tail golden bass” is in reference to the dark spot on the lower lobe of the caudal fin, which, along with other characters, distinguishes *Liopropoma santi* from the two other species of western Atlantic *Liopropoma* that have predominantly golden coloration, *Liopropoma aberrans* and *Liopropoma olneyi*.

#### Comparisons.

**Figure 5.**
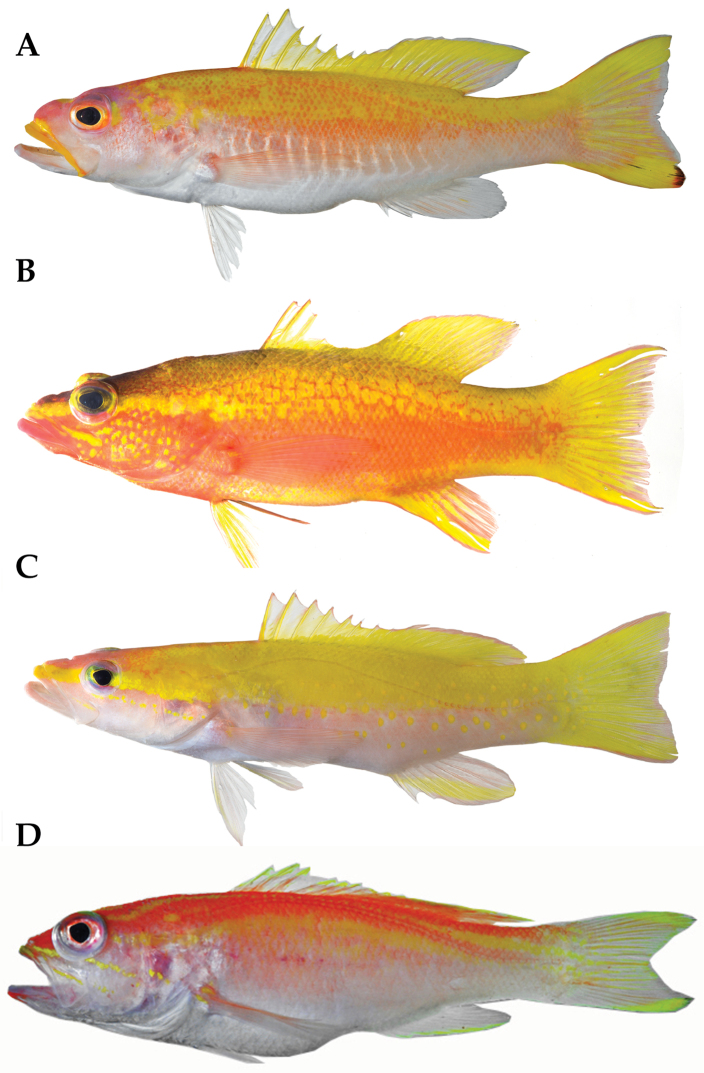
Comparison of the three species of “golden basses” off Curaçao and *Bathyanthias* sp. from Panama: **A** *Liopropoma santi* sp. n., USNM 426811, holotype, 116 mm SL, DNA #CUR 13280 **B** *Liopropoma aberrans*, USNM 426807, 102 mm SL, DNA #CUR 12226 **C** *Liopropoma olneyi*, USNM 426805, holotype, 84.3 mm SL, DNA #CUR 13200 **D** *Bathyanthias* sp., USNM 407791, 110 mm SL, DNA #MOC 11791.

Counts and measurements of the three western Atlantic “golden basses” collected off Curaçao, *Liopropoma santi*, *Liopropoma aberrans*, and *Liopropoma olneyi*, are given in Table 2, representative images of the three are provided in Figure 5, and a summary of major differences among them appears in Table 3. An image of a freshly collected specimen of a species of the related genus *Bathyanthias* is also included in Figure 5 for comparative purposes. *Liopropoma santi* is easily distinguished from the others by color in life, especially by the presence of a yellow or orange stripe externally on the upper lip, a series of white chevron-shaped markings on the ventral portion of the trunk, and the reddish-black blotch on the distal portion of the lower caudal-fin lobe. The last also visually distinguishes *Liopropoma santi* from *Liopropoma aberrans* and *Liopropoma olneyi* in preservative. *Liopropoma santi* is further distinguished from both of those species by having more dorsal-fin rays, more gill rakers on the first arch, and usually a larger eye (Table 2). From *Liopropoma aberrans*, *Liopropoma santi* is further distinguished by having more pectoral-fin rays, a narrower body at the dorsal-fin origin, a narrower caudal peduncle, longer fourth-sixth dorsal-fin spines, and a more shallow indentation in the spinous dorsal fin (Tables 2, 3).

**Table 2.** Selected counts and measurements for the type series of *Liopropoma santi* sp. n., *Liopropoma aberrans* from Curaçao, and *Liopropoma olneyi*. Measurements are in percentages of SL. Data for *Liopropoma aberrans* are from Curaçao specimens examined in this study, those for *Liopropoma olneyi* are from Baldwin and Johnson (2014).

	*Liopropoma santi*	*Liopropoma santi*	*Liopropoma santi*	*Liopropoma olneyi*	*Liopropoma aberrans*
Museum Catalog Numbers	USNM 426811 Holotype	USNM 426813 Paratype	USNM 414824 Paratype	See Appendix	See Appendix
SL (mm)	116	76.2	42.0	53.2-84.3	64.8-116
Dorsal Fin	VIII, 13	VIII, 13	VIII, 13	IX, 11	VIII, 12
Pectoral Fin	15	15	15	14-15	14
Gill Rakers on First Arch	6+14=20	6+14=20	6+15=21	5-6+12-13=17-19	5-6+11-13=17-18
Orbit diameter	9.4	10	12	7.8–9.4	7.4–8.7
Body depth at dorsal-fin origin	26	25	23	20–24	27–29
Least depth of caudal peduncle	13	13	11	13–15	16–17
Length of dorsal-fin spine IV	11	11	12	9.7–12	8.1–9.7
Length of dorsal-fin spine V	9.5	10	6.9	8.3–9.3	3.7–5.6
Length of dorsal-fin spine VI	8.2	7.9	6.9	7.3–8.9	3.6–5.6

**Table 3.** Summary of differences in morphology and depth ranges among the three golden-colored *Liopropoma* species off Curaçao.

Character	*Liopropoma santi* sp. n.	*Liopropoma olneyi*	*Liopropoma aberrans*
Relative body depth	Shallow (23–26% SL)	Shallow (20–24% SL)	Deeper (27–29% SL)
Dorsal fin indentation	Moderate (6^th^ spine 7–8% SL)	Weak (6^th^ spine 7–9% SL)	Strong (6^th^ spine 4–6% SL)
Dorsal-fin rays	VIII, 13	IX, 11	VIII, 12
Gill rakers on first arch	20–21	17–19	17–18
Orbit diameter (% SL)	9.4–12	7.8–9.4	7.4–8.7
White flank chevrons	yes	no	no
Body ground colors	yellow over white	yellow over white	yellow over orange
Yellow stripe through eye	no	yes	yes
Yellow-orange upper lip	yes	no	no
Yellow spots on body	no	adult & juvenile	juvenile only
Dark spot on lower caudal-fin lobe	yes	no	no
Depth range (m)	181–241	133–193	98–149

Baldwin and Johnson (2014) discussed the status of *Liopropoma aberrans*, which was described from a single specimen collected off Cuba in the 19^th^ century (Poey 1860) and redescribed from a single specimen collected off the Bahamas in the 20^th^ century (Robins 1967). They noted differences in the descriptions of color patterns of the two specimens and numbers of dorsal-fin rays (IX, 12 in Poey’s *Liopropoma aberrans*, VIII, 12 in Robins’ *Liopropoma aberrans*), and they questioned whether or not the two specimens represent the same species. Specimens of *Liopropoma aberrans* collected off Curaçao (“Curaçao *Liopropoma aberrans*”) share with the Bahamas *Liopropoma aberrans* the same dorsal-fin count, general body shape, and color pattern, although Baldwin and Johnson (2014) noted some differences in the color pattern. Curaçao *Liopropoma aberrans* have 17–18 gill rakers on the first arch (Table 2), whereas Robins (1967) reported 14 for the Bahamas specimen; however, as noted by Baldwin and Johnson (2014), Robins’ count only included the rudimentary pads on the upper limb. Examination of the Robins’ Bahamas specimen (UMML 22324) indicates that there are four rudimentary pads on the lower limb, and thus the total number of gill rakers on the first arch is 18.

Curaçao and Bahamas *Liopropoma aberrans*, however, appear to have different depth preferences, with Robins’ *Liopropoma aberrans* occurring deeper—229 m. At Curaçao, *Liopropoma aberrans* was collected between 98 and149 m and observed by us only within that depth range during nearly 100 submersible dives over a three-year period. This is unlikely to be due to effects of differences in habitat availability at the two locations, as *Liopropoma santi* and *Liopropoma olneyi* occur at deeper depths than *Liopropoma aberrans* at Curaçao.

Poey (1860) did not provide depth data or a gill-raker count for his 115-mm SL specimen from Cuba. Curaçao *Liopropoma aberrans* differs from the Cuban *Liopropoma aberrans* in dorsal-fin count and certain aspects of color pattern, but fish from those two sites share the presence of yellow spots on the cheek (sometimes lacking in juvenile Curaçao *Liopropoma aberrans*), spots that were not mentioned by Robins (1967) for the 112-mm SL Bahamas *Liopropoma aberrans*. The whereabouts of the holotype of *Liopropoma aberrans* are unknown (Eschmeyer 2013), and, in the absence of additional material from the type locality for comparative purposes, we follow Baldwin and Johnson (2014) in tentatively recognizing the specimens from Cuba, Bahamas, and Curaçao as *Liopropoma aberrans*. As noted by Baldwin and Johnson (2014), a digitized copy of a color photograph of a specimen of *Liopropoma aberrans* from Jamaica taken and provided by Patrick Colin shows a color pattern nearly identical to that of Curaçao *Liopropoma aberrans*. Should Poey’s *Liopropoma aberrans* prove to be distinct from specimens from the Bahamas, Curaçao, and Jamaica, one or more new species will need to be recognized.

*Liopropoma santi* differs from Poey’s and Robins’ *Liopropoma aberrans* in number of dorsal-fin rays (VIII, 13 vs. IX, 12 and VIII, 12, respectively) and shape of dorsal fin (with only a moderate indentation in spinous dorsal fin in *Liopropoma santi*, deep indentation in the others). It further differs from Robins’ *Liopropoma aberrans* in numbers of pectoral-fin rays (15 vs. 14) and gill rakers on the first arch (20–21 vs. 17–18), and color pattern (presence of diagnostic color features of *Liopropoma santi*–see Diagnosis–vs. absence). From other western Atlantic *Liopropoma* (*Liopropoma carmabi* [Randall 1963], *Liopropoma eukrines* [Starck and Courtenay 1962], *Liopropoma mowbrayi*[Woods and Kanazawa 1951], *Liopropoma rubre* Poey 1861), *Liopropoma santi* differs most notably in color pattern (Fig. 1) and in having VIII, 13 dorsal-fin rays (vs. VIII, 12 in all except one specimen of *Liopropoma carmabi* with VIII, 13–Table 4).

**Table 4.** Dorsal-fin counts of western Atlantic Liopropomini fishes. Data for *Bathyanthias atlanticus*, *Bathyanthias cubensis*, and *Bathyanthias mexicanus* are from Schultz (1958); for *Liopropoma aberrans* (Cuba) Poey (1860); for *Liopropoma aberrans* (Bahamas) Robins (1967); for *Liopropoma carmabi*, *Liopropoma eukrines*, *Liopropoma mowbrayi*, *Liopropoma rubre* Randall (1963); and for *Liopropoma olneyi* Baldwin and Johnson (2014).

	SPINES		SOFT RAYS				
	VIII	IX	11	12	13	14	15
*Bathyanthias atlanticus*	+					+	
*Bathyanthias cubensis*	+				+		
*Bathyanthias mexicanus*	+					+	+
*Bathyanthias roseus*^1^	+					+	
*Liopropoma aberrans* (Curaçao)	+			+			
*Liopropoma aberrans* (Cuba)		+		+			
*Liopropoma aberrans* (Bahamas)	+			+			
*Liopropoma carmabi*	+			+	+		
*Liopropoma eukrines*	+			+			
*Liopropoma mowbrayi*	+			+			
*Liopropoma olneyi*		+	+				
*Liopropoma rubre*	+			+			
*Liopropoma santi* sp. n.	+				+		

^1^ As noted by Baldwin and Johnson (1993), Günther (1880) gave IX, 14 as the dorsal-fin count for *Bathyanthias roseus*, but their examination of a radiograph of the type specimen indicates that it has VIII dorsal spines.

**Figure 6.**
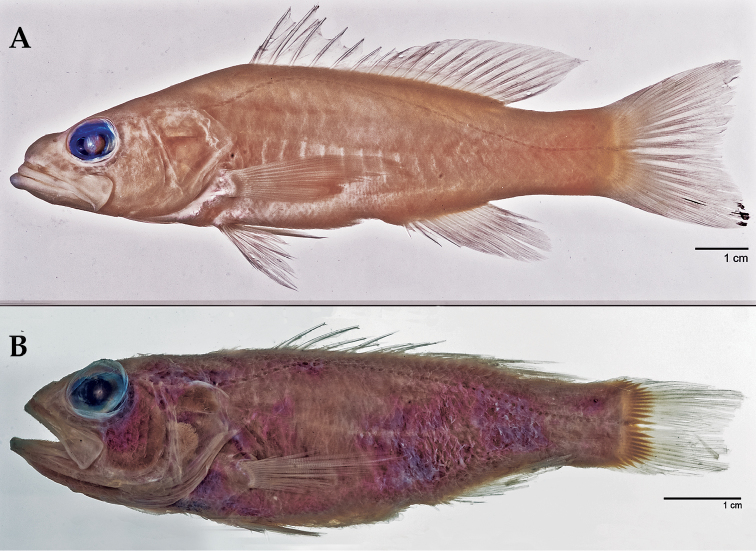
Comparison of *Liopropoma* and *Bathyanthias*: **A** *Liopropoma santi* sp. n., USNM 426811, holotype, 116 mm SL (photographed after preservation) **B** *Bathyanthias cubensis* (Schultz 1958), USNM 158138, holotype, 80.3 mm SL. Photos by Sandra Raredon.

Counts of *Liopropoma santi* closely match those of *Bathyanthias cubensis* (Schultz 1958) in having VIII, 13 dorsal-fin rays; III, 8 anal-fin rays; 15 pectoral-fin rays; and 20–21 gill rakers on the first arch. *Liopropoma santi* has 47–49 lateral-line scales, whereas *Bathyanthias cubensis* has 46–47. The two species are otherwise very different. *Liopropoma santi* has a shallower trunk (body depth 23–26% SL and caudal-peduncle depth 11–13% SL in *Liopropoma santi* vs. 28–32% SL and 14–15% SL, respectively, in *Bathyanthias cubensis*–Schultz 1958), and *Liopropoma santi* has a single blotch of dark pigment on the distal portion of the lower caudal-fin lobe vs. dark pigment on the distal ends of all caudal-fin rays. Like other species of *Bathyanthias*, the dorsal profile of the head in *Bathyanthias cubensis* is convex (vs. usually straight in *Liopropoma*—although there may be a bump on the snout and the profile may be slightly convex in large specimens of *Liopropoma*); there is little indentation in the margin of the spinous dorsal fin (vs. larger indentation); the posteroventral corner of the maxilla has a weakly developed hook-like process (vs. well developed in *Liopropoma*—see Randall and Taylor [1988] and Baldwin and Johnson [1993]); and in *Bathyanthias*, the anterior portion of the lateral line is broadly curved over the pectoral fin (vs. sharply curved in *Liopropoma*). Differences between *Liopropoma santi* and *Liopropoma cubensis* can be seen in Figure 6, and the generic characters listed above can be seen in Figures 5 and 6. The depth range of *Bathyanthias cubensis* is greater than that of *Liopropoma santi*, 183–411 m vs. 182–241 m.

## Discussion and conclusions

**Figure 7.**
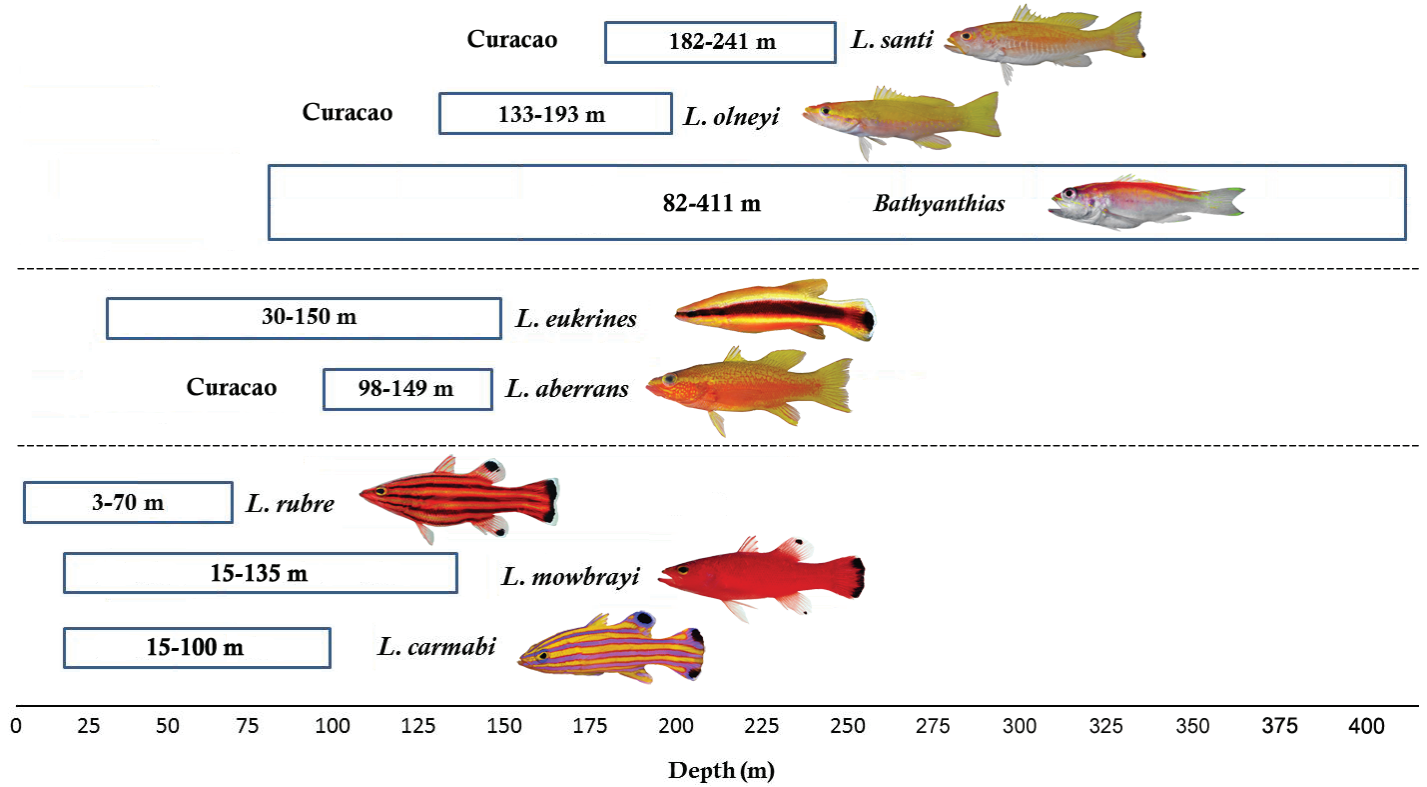
Depth distributions of western Atlantic *Liopropoma* and *Bathyanthias* species that were included in the phylogenetic analysis (see Fig. 8). Photographs of *Liopropoma rubre* and *Liopropoma mowbrayi* by James Van Tassell and Ross Robertson.

**Figure 8.**
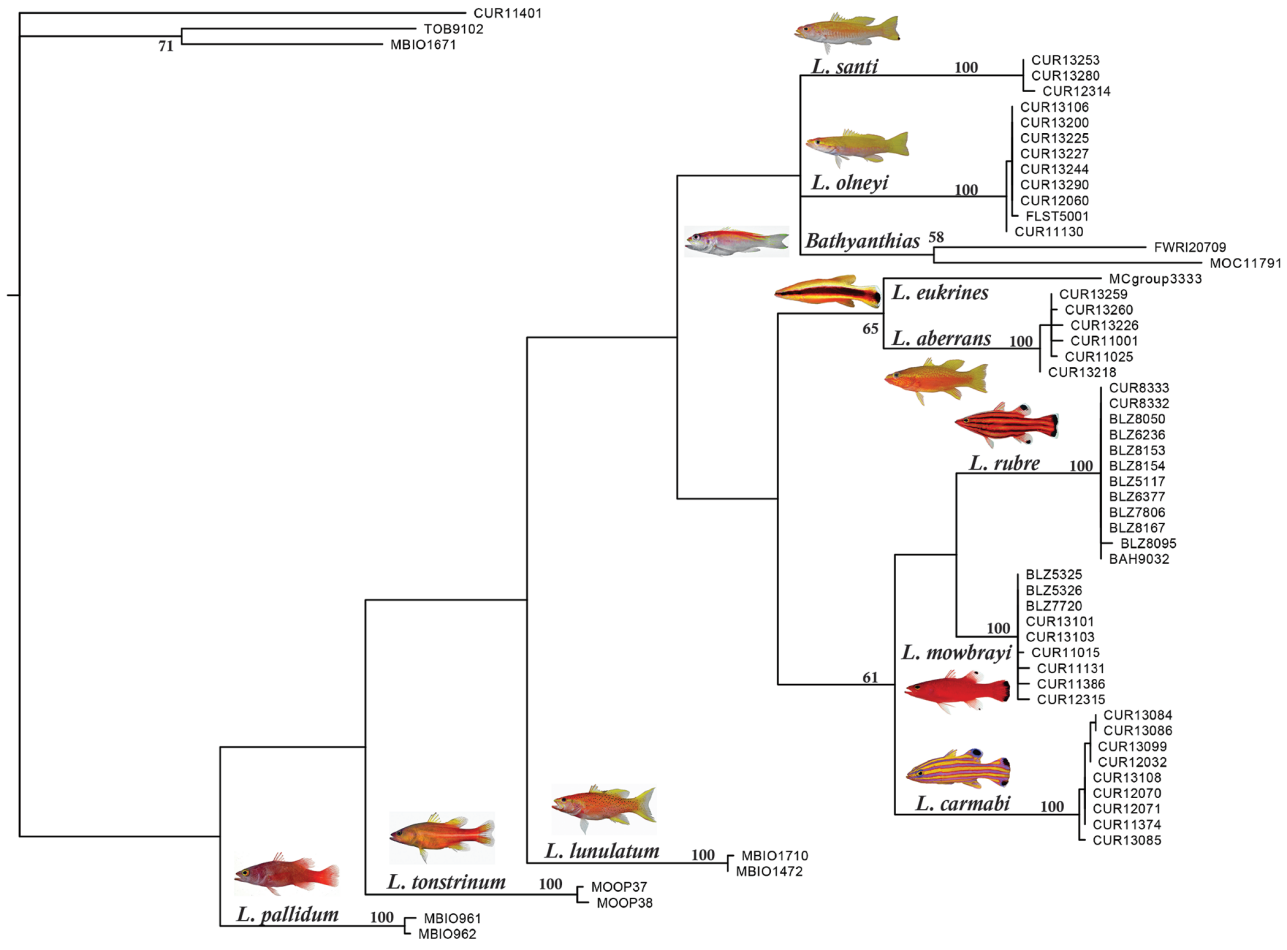
The strict consensus of a maximum parsimony analysis of the COI region among western Atlantic *Liopropoma* and related taxa. The tree was rooted on *Scorpaena plumieri*, (CUR11401), and the non-liopropomin serranids *Rypticus carpenteri* (TOB9102) and *Grammistes sexlineatus* (MBIO1671) were included as additional outgroups. Photographs of *Liopropoma rubre* and *Liopropoma mowbrayi* by James Van Tassell and Ross Robertson; photos of *Liopropoma pallidum* and *Liopropoma lunulatum* by Jeffrey Williams (from Encyclopedia of Life); photo of *Liopropoma tonstrinum* by Richard Winterbottom (from Encyclopedia of Life).

A combination of morphological and genetic differences supports the recognition of *Liopropoma santi* as a valid new species of *Liopropoma*. *Liopropoma santi* inhabits depths of 182-241 m off Curaçao, making it the deepest known *Liopropoma* species in the western Atlantic (Fig. 7). The shallower portion of its depth range overlaps the deeper portion of the depth range of *Liopropoma olneyi* (133–193 m), but with the exception of Robins’ (1967) specimen of *Liopropoma aberrans* from the Bahamas (229 m), no other western Atlantic *Liopropoma* species occur within the depth range of *Liopropoma santi*. A preliminary phylogeny of western Atlantic *Liopropoma* based on parsimony analysis of the COI data is shown in Figure 8. In that phylogeny, the three species that inhabit depths of 3–135 m (*Liopropoma rubre*, *Liopropoma carmabi*, and *Liopropoma mowbrayi*) form a monophyletic group that is sister to a clade comprising two species that inhabit depths of 30–150 m (*Liopropoma eukrines* and *Liopropoma aberrans* from Curaçao). Those clades combined are sister to a clade comprising the deepest western Atlantic *Liopropoma* (*Liopropoma olneyi* and *Liopropoma santi*, 133–241 m) plus two species of the genus *Bathyanthias* (*Bathyanthias mexicanus*[Schultz 1958] and a putative new species from Panama) that were collected at 143–259 m. Two additional species of *Bathyanthias*, *Bathyanthias atlanticus* [Schultz 1958] and *Bathyanthias cubensis* (not available for inclusion in the molecular phylogenetic analysis), are known from 82–411 m, and the depth range of non-Curacao *Liopropoma aberrans* (also not available for inclusion in the phylogenetic analysis) is 89–230 m (Robins 1967, Ocean Biogeographic Information System [OBIS] - http://www.iobis.org/, Fishnet 2 - http://www.fishnet2.net/).

The COI data provide excellent support for the monophyly of species of western Atlantic *Liopropoma* but poor support for clades within the genus (see bootstrap values in Fig. 8). Nevertheless, the strict consensus (Fig. 8) suggests that western Atlantic liopropomins are monophyletic with respect to Indo-Pacific *Liopropoma* (*Liopropoma lunulatum*, *Liopropoma tonstrinum*, and *Liopropoma pallidum* in Fig. 8). A more robust phylogenetic hypothesis is needed that is derived from additional genes and more Indo-Pacific species of *Liopropoma*, but the COI data suggest a relationship between depth and monophyletic clades in western Atlantic Liopropomini that warrants further investigation. Members of the three clades of western Atlantic liopropomins identified in the phylogeny show a tendency to occupy different depth strata (3–135 m, 30–150 m, and 82–411 m). Based on our few specimens, it appears that *Liopropoma santi* has larger eyes than its sister species, *Liopropoma olneyi* (Table 3), which may represent an adaptation allowing *Liopropoma santi* to extend its range to greater depths. Among the three golden basses at Curaçao (*Liopropoma aberrans*, *Liopropoma olneyi*, *Liopropoma santi*), *Liopropoma aberrans* has the shallowest range and shows a tendency to have the smallest eyes (Table 3). Adaptation to life at different depths may have been involved in the speciation of this co-occuring species group. It may also be involved in the divergence between *Liopropoma mowbrayi* and *Liopropoma rubre*, which represent sister species that show only partial overlap in their depth ranges (Fig. 7) but broadly overlapping geographic ranges that incorporate most of the Caribbean and adjacent areas. Adaptation to use of different depth strata may also have been involved in the initial diversification of western Atlantic liopropomins into three clades that now occupy the same geographic area. Such parapatric ecological speciation, in which species diverge along environmental gradients, has been proposed for other marine fishes including *Halichoeres* (Rocha et al. 2005) and *Sebastes* (Ingram 2011). In *Sebastes*, Ingram (2011) found a strong signal of speciational evolution in depth habitats and in traits apparently related to life at different depths, such as eye size.

Conversely, the sister species *Liopropoma eukrines* and *Liopropoma aberrans* overlap substantially in depth range but show a significant amount of geographic separation: *Liopropoma eukrines* is largely restricted to the Gulf of Mexico and southeastern USA, whereas *Liopropoma aberrans* is primarily Caribbean. However, there is one inconsistency in this pattern of either geographic or depth segregation among members of the same clade: *Liopropoma carmabi* and both species in its sister group, *Liopropoma rubre* and *Liopropoma mowbrayi*, have both geographic- and depth ranges that broadly overlap. Liopropomins have pelagic larvae, and allopatric speciation might be facilitated by larval dispersal to new areas. Possibly both ecological and allopatric speciation have occurred in the group, but, if so, more information on depth and geographic distributions, morphological traits associated with life at different depths, and evolutionary relationships is needed to estimate their relative roles. Depth and morphological information for the three members of the *Liopropoma rubre* clade collected at the same geographic location would be highly relevant in this regard. At Puerto Rico all three species in that clade occur on the same mesophotic reefs, where they reach the same maximum depth (Bejarano et al 2014). At Curaçao Adriaan Schrier, who operates Substation Curaçao, has been actively collecting all three species for many years using a combination of traditional SCUBA, mixed gas SCUBA, and the *Curasub*. He provided (personal communicatinetic differences supports the recognition of location: *Liopropoma rubre* occurs at 12–55 m, *Liopropoma mowbrayi* at 12–135m, and *Liopropoma carmabi* at 25–100m. He also noted that while *Liopropoma mowbrayi* and *Liopropoma carmabi* are found in areas with small-scale coral and rock shelter and rubble, *Liopropoma rubre* is restricted to caves in large scale coral structures and is much more secretive than the other two species. These observations indicate that members of the *Liopropoma rubre* clade show some degree of depth segregation within a site, as well as microhabitat segregation.

The phylogeny (Fig. 8) further suggests the need to reanalyze generic relationships within the Liopropomini, as *Bathyanthias* is embedded within western Atlantic *Liopropoma*. Morphologically, *Liopropoma santi*, *Liopropoma olneyi*, and *Bathyanthias* differ from other western Atlantic *Liopropoma* in having a smaller indentation in the margin of the dorsal fin, and those liopropomins lack body stripes and have similar pale orange/yellow/rose coloration. Four species of *Bathyanthias* have been described–*Bathyanthias atlanticus* (Schultz, 1860), *Bathyanthias cubensis* (Schultz, 1860), *Bathyanthias mexicanus* (Schultz, 1860), and *Bathyanthias roseus* Günther 1880. Of those, only *Bathyanthias mexicanus* from the Gulf of Mexico (FWRI 20709) was available for inclusion in our phylogenetic analysis. The other *Bathyanthias* species included, which may represent an undescribed species, is from Central America - Panama (USNM 407791, MOC 11791). Its combination of dorsal-, pectoral, lateral-line, and gill-raker counts do not match any other known species of *Bathyanthias*.

Of the three western Atlantic species of *Liopropoma* with depth distributions entirely below depths accessible using conventional scuba gear—*Liopropoma aberrans*, *Liopropoma olneyi*, and *Liopropoma santi*—two have been discovered only recently through submersible diving to 300 m off Curaçao in the southern Caribbean (*Liopropoma olneyi* and *Liopropoma santi*). More exploration of western Atlantic tropical mesophotic and other deep-reef depths is needed to fully document fish diversity even in well-studied taxonomic groups such as the Serranidae.

## Comparative material

Specimens, color images, or both, were examined of all western Atlantic liopropomin material listed in the Appendix. The following non-Curaçao *Liopropoma aberrans* material was examined: UF 222324, 1 specimen, Bahamas; UF 230721, 1, Jamaica; UF 230254, 1, French Guiana.

## Supplementary Material

XML Treatment for Liopropoma santi

## Appendix

**Table:** Links between DNA voucher specimens, GenBank accession numbers, and cytochrome c oxidase subunit I (COI) sequences of *Liopropoma santi* sp. nov., related Liopropomini, and outgroup taxa.

Catalog Number/DNA Number	GenBank No.	GenSeq Designation
*Liopropoma santi* sp. n.		
USNM 426811, CUR 13253, Holotype	KJ526147	Geneseq-1 COI
USNM 426813, CUR 13280, Paratype	KJ526148	genseq-2 COI
USNM 414824, CUR 12314, Paratype	KJ526146	genseq-2 COI
*Liopropoma olneyi*		
USNM 426805, CUR 13200, Holotype	KF770874	genseq-1 COI
USNM 406130, CUR 11130, Paratype	KF770856	genseq-2 COI
USNM 414828, CUR 12060, Paratype	KF770862	genseq-2 COI
USNM 426808, CUR 13225, Paratype	KF770876	genseq-2 COI
USNM 426809, CUR 13227, Paratype	KF770878	genseq-2 COI
USNM 426810, CUR 13244, Paratype	KF770879	genseq-2 COI
USNM 426815, CUR 13290, Paratype	KF770882	genseq-2 COI
USNM 422698, CUR13106, Paratype	KF770872	genseq-2 COI
USNM 426868, FLST 5001, Paratype (larva)	KF770883	
*Liopropoma aberrans*		
USNM 406001, CUR 11001	KF770853	genseq-4 COI
USNM 406025, CUR 11025	KF770855	genseq-4 COI
USNM 426806, CUR 13218	KF770875	genseq-4 COI
USNM 426807, CUR 13226	KF770877	genseq-4 COI
USNM 426814, CUR 13259	KF770880	genseq-4 COI
USNM 426812, CUR 13260	KF770881	genseq-4 COI
*Liopropoma carmabi*		
USNM 406374, CUR 11374	KF770858	genseq-4 COI
USNM 414825, CUR 12032	KF770861	genseq-4 COI
USNM 414826, CUR 12070	KF770863	genseq-4 COI
USNM 414827, CUR 12071	KF770864	genseq-4 COI
USNM 413959, CUR 13084	KF770866	genseq-4 COI
USNM 413960, CUR 13085	KF770867	genseq-4 COI
USNM 413961, CUR 13086	KF770868	genseq-4 COI
USNM 422694, CUR 13099	KF770869	genseq-4 COI
USNM 422687, CUR 13108	KF770873	genseq-4 COI
*Liopropoma eukrines*		
SIO 01-11, MCgroup 3333	KF770885	genseq-4 COI
*Liopropoma mowbrayi*		
USNM 420350, BLZ 5325	JQ840569	genseq-4 COI
USNM 420349, BLZ 5326	JQ840570	genseq-4 COI
BLZ 7720 (photo voucher only)	JQ841243	genseq-5 COI
USNM 406015, CUR 11015	KF770854	genseq-4 COI
USNM 406131, CUR 11131	KF770857	genseq-4 COI
USNM 406386, CUR 11386	KF770859	genseq-4 COI
USNM 414815, CUR 12315	KF770865	genseq-4 COI
USNM 422684, CUR 13101	KF770870	genseq-4 COI
USNM 422675, CUR 13103	KF770871	genseq-4 COI
*Liopropoma rubre*		
USNM 414697, BAH 9032	KF770852	genseq-4 COI
USNM 419340, BLZ 5117	JQ840571	genseq-4 COI
USNM 416331, BLZ 6236	JQ840899	genseq-4 COI
USNM 416379, BLZ 6377	JQ840900	genseq-4 COI
USNM 416009, BLZ 7806	JQ841244	genseq-4 COI
USNM 415207, BLZ 8050	JQ841640	genseq-4 COI
USNM 415226, BLZ 8095	JQ841637	genseq-4 COI
USNM 415180, BLZ 8153	JQ841638	genseq-4 COI
USNM 415181, BLZ 8154	JQ841641	genseq-4 COI
USNM 415244, BLZ 8167	JQ841639	genseq-4 COI
USNM 414498, CUR 8332	JQ842192	genseq-4 COI
USNM 414499, CUR 8333	JQ842193	genseq-4 COI
*Liopropoma lunulatum* (Pacific)		
MBIO 1710 (no photo or specimen voucher)	JQ431889	no classification
MNHN 2008-1023, MBIO 1472	JQ431888	genseq-4 COI
*Liopropoma tonstrinum* (Pacific)		
USNM 425632, MOOP37	KJ526149	genseq-4 COI
USNM 425630, MOOP38	KJ526150	genseq-4 COI
*Liopropoma pallidum* (Pacific)		
MNHN 2009-0793, MBIO 961	JQ431890	genseq-4 COI
MNHN 2009-0794, MBIO 962	JQ431891	genseq-4 COI
*Bathyanthias mexicanus*		
FWRI 20709 (DNA number same)	KF770884	genseq-4 COI
*Bathyanthias* sp.		
USNM 407791, MOC 11791	KF770886	genseq-4 COI
Outgroup Taxa		
*Grammistes sexlineatus*		
MNHN 2008-1105, MBIO 1671	JQ431776	genseq-4 COI
*Rypticus carpenteri*		
USNM 401296, TOB 9102	JN828097	genseq-4 COI
*Scorpaena plumieri*		
USNM 406401, CUR 11401	KF770860	genseq-4 COI

## References

[B1] Baldwin CC Johnson GD (2014) Connectivity across the Caribbean Sea: DNA barcoding and morphology unite an enigmatic fish larva from the Florida Straits with a new species of sea bass from deep reefs off Curaçao. PLoS ONE 9(5): . doi: 10.1371/journal.pone.0097661 10.1371/journal.pone.0097661 PMC4019605 24825118

[B2] Baldwin CC Johnson GD (1993) Phylogeny of the Epinephelinae (Teleostei: Serranidae). Bulletin of Marine Science 22: 240-283

[B3] Baldwin CC Weigt LA (2012) A new species of soapfish (Teleostei: Serranidae: *Rypticus*), with redescription of *R. subbifrenatus* and comments on the use of DNA barcoding in systematic studies. Copeia 2012: 23-36. doi: 10.1643/CG-11-035

[B4] Baldwin CC Robertson DR (2013) A new *Haptoclinus* blenny (Teleostei, Labrisomidae) from deep reefs off Curacao, southern Caribbean, with comments on relationships of the genus. ZooKeys 306: 71-81. doi: 10.3897/zookeys.306.5198 23794919 10.3897/zookeys.306.5198 PMC3689043

[B5] Bejarano L Appeldorn RS Nemeth M (2014) Fishes associated with mesophotic coral ecosystems at La Parguera, Puerto Rico. Coral Reefs. doi: 10.1007/s00338-014-1125-6

[B6] Betancur RR Broughton RE Wiley EO Carpenter K López JA Li C Holcroft NI Arcila D Sanciangco M Cureton JC II Zhang F Buser T Campbell MA Ballesteros JA Roa-Varon A Willis S Borden WC Rowley T Reneau PC Hough DJ Lu G Grande T Arratia G Ortí G (2013) The tree of life and a new classification of bony fishes. PLoS Currents Tree of Life. 2013 April 18. doi: 10.1371/currents.tol.53ba26640df0ccaee75bb165c8c26288 10.1371/currents.tol.53ba26640df0ccaee75bb165c8c26288 PMC3644299 23653398

[B7] Chakrabarty P Warren M Page LM Baldwin CC (2013) GenSeq: An updated nomenclature and ranking for genetic sequences from type and non-type sources. Zookeys 346: 29-41 24223486 10.3897/zookeys.346.5753 PMC3821064

[B8] Eschmeyer WN (Ed) (2013) Catalog of fishes: genera, species, references. http://research.calacademy.org/research/ichthyology/catalog/fishcatmain.asp [Accessed 10 Dec 2013]

[B9] Günther A (1880) Report on the shore fishes procured during the voyage of H. M. S. Challenger in the years 1873-1876. In: Report on the scientific results of the voyage of H.M.S. Challenger during the years 1873-76. Zoology. v. 1 (pt 6): 1–82, Pls. 1–32

[B10] Hubbs CL Lagler KF (1958) Fishes of the Great Lakes region. University of Michigan Press Ann, Arbor, Michigan, 213 pp

[B11] Ingram T (2011) Speciation along a depth gradient in a marine adaptive radiation. Proceedings of the Royal Society B 278: 613-618 20810434 10.1098/rspb.2010.1127 PMC3025674

[B12] Johnson GD (1983) *Niphon spinosus*: a primitive epinepheline serranid, with comments on the monophyly and interrelationships of the Serranidae. Copeia: 777–787

[B13] Kimura M (1980) A simple method for estimating evolutionary rates of base substitutions through comparative studies of nucleotide sequences. Journal of Molecular Evolution 16: 111-120 7463489 10.1007/BF01731581

[B14] Near TJ Dornburg A Eytan RI Keck BP Smith WL Kuhn KL Moore JA Price SA Burbrink FT Friedman M Wainwright PC (2013) Phylogeny and tempo of diversification in the superradiation of spiny-rayed fishes. Proceedings of the National Academies of Science 110: 12738-12743. doi: 10.1073/pnas.1304661110 10.1073/pnas.1304661110 PMC3732986 23858462

[B15] Poey Y (1860) Poissons de Cuba, espèces nouvelles. Memorias Sobre la Historia Natural de la Islad de Cuba 2: 115-335

[B16] Poey Y (1861) Memorias sobra la historia natural de la Isla de Cuba, acompañadas de sumarios Latinos y extractos en Francés. Tomo 2 La Habana 2: 1–96 (1858), 97–336 (1860), 337–442 (1861), Pls. 1–19

[B17] Randall JE (1963) Three new species and six new records of small serranoid fishes from Curaçao and Puerto Rico. Studies of the Fauna of Curacao and other Caribbean Islands 19: 77-110

[B18] Randall JE Taylor L (1988) Review of the Indo-Pacific fishes of the serranid genus *Liopropoma*, with descriptions of seven new species. Indo-Pacific Fishes 16: 1-47

[B19] Robins CR (1967) The status of the serranid fish *Liopropoma aberrans*, with the description of a new, apparently related genus. Copeia 1967: 591-595. doi: 10.2307/1442237

[B20] Rocha LA Robertson DR Roman J Bowen BW (2005) Ecological speciation in tropical reef fishes. Proceedings of the Royal Society B 272: 573-579. doi: 10.1098/2004.3005 15817431 10.1098/2004.3005 PMC1564072

[B21] Sabaj Pérez MH (Ed) (2012) Standard symbolic codes for institutional resource collections in herpetology and ichthyology: an Online Reference. Version 3.0. American Society of Ichthyologists and Herpetologists, Washington, DC http://www.asih.org/ [accessed on 23 February 2012]

[B22] Saitou N Nei M (1987) The neighbor-joining method: a new method for reconstructing phylogenetic trees. Molecular Biological Evolution 14: 406-425 10.1093/oxfordjournals.molbev.a040454 3447015

[B23] Schultz LP (1958) Three new serranid fishes, genus *Pikea*, from the western Atlantic. Proceedings of the United States National Museum 108: 321-329. doi: 10.5479/si.00963801.108-3405.321

[B24] Seutin G Bagley P White BN (1991) Preservation of avian blood and tissue samples for DNA analyses. Canadian Journal of Zoology 69: 82-90. doi: 10.1139/z91-013

[B25] Smith WL Craig MT (2007) Casting the percomorph net widely: The importance of broad taxonomic sampling in the search for the placement of serranid and percid fishes. Copeia: 35–55. doi: 10.1643/0045-8511(2007)7[35:CTPNWT]2.0.CO;2

[B26] Stark WA II Courtenay WR Jr. (1962) *Chorististium eukrines*, a new serranid fish from Florida, with notes on related species. Proceedings of the Biological Society of Washington 75: 159-167

[B27] Swofford D (2002) PAUP*: phylogenetic analysis using parsimony (*and other methods). Sinauer Associates, Sunderland, Massachusetts

[B28] Thunberg CP (1792) Åtskillige förut okánde Fiskar af abborslágtet. Kongliga Vetenskaps Akademiens nya Handlingar, Stockholm v. 13 (for 1792): 141–143, Pl. 5

[B29] Weigt LA Driskell AC Baldwin CC Ormos A (2012) DNA Barcoding Fishes. In: Kress WJ Erickson DL (Eds) DNA Barcodes: Methods and Protocols.Methods in Molecular Biology 858: 109-126 22684954 10.1007/978-1-61779-591-6_6

[B30] Woods LP Kanazawa RH (1951) New species and new records of fishes from Bermuda. Fieldiana Zoology 31: 629-644

